# Meningitis-retention syndrome: a review and update of an unrecognized clinical condition

**DOI:** 10.1007/s10072-023-06704-0

**Published:** 2023-03-03

**Authors:** Francesco Pellegrino, Elisa Funiciello, Giulia Pruccoli, Erika Silvestro, Carlo Scolfaro, Federica Mignone, Aba Tocchet, Luca Roasio, Silvia Garazzino

**Affiliations:** 1 Department of Pediatric and Public Health Sciences, Regina Margherita Children’s Hospital, Postgraduate School of Pediatrics, University of Turin, Piazza Polonia 64, Turin, Italy; 2 Department of Pediatric and Public Health Sciences, Infectious Diseases Unit, Regina Margherita Children’s Hospital, Turin, Italy; 3 Child and Adolescent Neurology and Psychiatry Division, Città Della Salute E Della Scienza Hospital, University of Turin, Turin, Italy; 4 Pediatric Department, E. Agnelli Hospital, Via Brigata Cagliari 39, Pinerolo, TO Italy

**Keywords:** Meningitis-retention syndrome, Urinary retention, Acute disseminated encephalomyelopathy, Aseptic meningitis

## Abstract

**Objectives:**

We summarized the clinical and radiological characteristics of meningitis-retention syndrome (MRS), its therapeutic options, and urological outcome, to better understand the pathogenesis of this syndrome and to evaluate the effectiveness of corticosteroids in reducing the period of urinary retention.

**Methods:**

We reported a new case of MRS in a male adolescent. We also reviewed the previously 28 reported cases of MRS, collected from inception up to September 2022.

**Results:**

MRS is characterized by aseptic meningitis and urinary retention. The mean length of the interval between the onset of the neurological signs and the urinary retention was 6.4 days. In most cases, no pathogens were isolated in cerebrospinal fluid, except for 6 cases in which Herpesviruses were detected. The urodynamic study resulted in a detrusor underactivity, with a mean period for urination recovery of 4.5 weeks, regardless of therapies.

**Discussion:**

Neurophysiological studies and electromyographic examination are not pathological, distinguishing MRS from polyneuropathies. Although there are no encephalitic symptoms or signs, and the magnetic resonance is often normal, MRS may represent a mild form of acute disseminated encephalomyelitis, without radiological detectable medullary involvement, due to the prompt use of steroids. It is believed that MRS is a self-limited disease, and no evidence suggests the effectiveness of steroids, antibiotics, and antiviral treatment in its clinical course.

## Introduction

Acute urinary retention (AUR) is a common urological emergency, presenting as a sudden inability to voluntarily void, and is typically associated with lower abdominal pain. Although the most common cause is benign prostatic hyperplasia (BPH), other causes include urinary infections, constipation, sacral spinal cord diseases, such as Guillain-Barrè syndrome, cerebral demyelinating diseases, such as acute disseminated encephalomyelitis (ADEM), and aseptic meningitis (AM). Rarely, acute urinary retention has also been reported as an adverse drug effect or as a post-surgery consequence [1].

Meningitis-retention syndrome (MRS) is a peculiar condition characterized by aseptic meningitis (AM), typically without any clear causative agent, associated with acute urinary retention [2]. The typical symptoms and neurological signs of aseptic meningitis are usually mild or absent, so the predominant symptom often turns out to be isolated acute urinary retention. Although several cases are reported in the literature, MRS actual prevalence is underestimated. These factors make an early diagnosis of MRS difficult. In the present study, we reported a new case of MRS in a male adolescent referred to our hospital, and we reviewed the previously reported individuals with MRS. This is the first review that described and compared all these cases of MRS, to summarize our knowledge of this rare case of acute urinary retention.

## Case description

A previously healthy 15-year-old male adolescent presented to the Emergency Department because of leg weakness and urinary retention for 24 h. He had been experiencing high fever accompanied by headache for a week, and he was treated with oral amoxicillin at home. On admission, the patient was febrile but fully conscious, and his mental status was not altered (Glasgow Coma Scale score of 15). Neurological examination revealed lower back pain associated with referred apparent sacral paraesthesia. A transurethral catheterization was performed, and 1000 cc of urine was removed. Blood test results were normal, inflammation markers (C-reactive protein and procalcitonin) were negative, and no abnormalities were noted in urinalysis. All serological, molecular, and culture tests performed showed no ongoing infection.

**Fig. 1:**
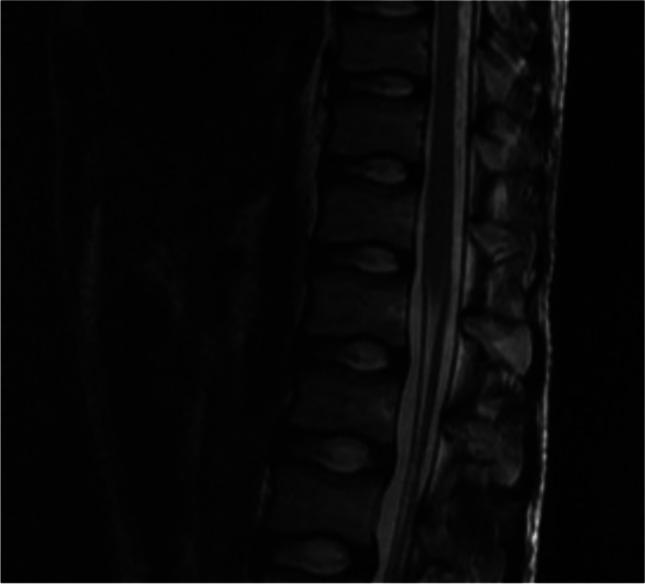
First RMN showing leptomeningeal thickening in T2-weighted image

A lumbar puncture was performed, and cerebrospinal fluid (CSF) examination showed mononuclear dominant lymphocytic pleocytosis (173 cells/mm3), increased protein content (158 mg/dl), and slightly decreased glucose levels (41 mg/dl). Cultural tests performed on CSF were negative, while isoelectric-focusing and k-index were positive. On magnetic resonance imaging (MRI) of the brain and the spinal cord, meningeal thickening and leptomeningeal enhancement of the conus and cauda equina were evident, without medullary involvement (Fig. 1).

The electroencephalogram (EEG) was not informative, and the electromyography was normal.

At hospitalization, broad-spectrum antibiotics (ceftriaxone 2 g/die and vancomycin 400 mg × 4/die) and antivirals (acyclovir 500 mg × 3/die) were promptly started in association with corticosteroids (9 mg × 3/die). The urodynamic tests showed an areflexic detrusor. During bladder filling, he felt a first sensation to void at 250 ml and a strong desire to void at 460 ml, but the sphincter EMG activity disappeared, and detrusor contraction was not visible.

On day 14 after the admission, he repeated an MRI that showed stable radiological findings; a second lumbar puncture was performed with a similar outcome to the previous one, including the culture test. However, due to the persistence of urinary retention, a third MRI was performed a week later, showing a less intense leptomeningeal enhancement of the cauda.

In the suspicion of immune-mediated meningitis, antibiotics and antivirals were suspended (total duration of therapy: 21 days), and corticosteroids were continued for another week (total duration of therapy 28 days), with persistent apyrexia and resumption of spontaneous urination. Meanwhile, the antibodies against gangliosides (GD1a, GD1b, GQ1b, GM1, and GM2) were made and resulted negative. He was discharged home after 30 days of hospitalization.

After a month, the patient repeated an MRI that did not show any radiological sign of pathology, and posterior tibial nerve somatosensory-evoked potential (SEP), which confirmed a residual bilateral delay in latency of N22-P40. The patient is still asymptomatic, with no residual neurological deficit, and in good overall condition. He will continue his neurological follow-up in our hospital to monitor SEP.

## Materials and methods

In this retrospective study, we conducted a systematic literature review of MEDLINE, EMBASE, PubMed, Orphanet, and the Cochrane Library databases to identify studies describing cases of MRS, in order to characterize the frequency, clinical symptoms, urodynamic findings, and management of this syndrome.

The selection and search of the articles were done in accordance with the PRISMA guidelines. All the selected abstracts and papers were read in full text, when available. This electronic search strategy was augmented by a manual examination of references cited in articles, recent reviews, editorials, and meta‐analyses.

We included all clinical studies, including case reports describing MRS patients. Three reviewers systematically searched PubMed and Embase and collected from inception up to September 2022 for any clinical evidence for MRS.

No restrictions were imposed on the language (also non-English literature was included), study period, or type of studies, including case reports that described patients with MRS.

Details of the criteria established a priori were as follows.

- Population: only human patients with diagnosis of MRS were included, with no restrictions on age or other demographics.
- Outcomes: only patients were included that had at least one of the two following criteria:
- Patients diagnosed with aseptic meningitis, who had symptoms or signs of meningeal inflammation without any clinical and radiological evidence of cerebral or medullary parenchymal involvement and if their cultures were negative
- Acute urinary retention that requires catheterization, appearing simultaneously or few days after occurrence of AM
- Study design: all study designs were included. Case reports and letters to the editor were included, if all other criteria for inclusion were satisfied.

The following data were collected from the studies retrieved: first author, year of publication, type of article, number of cases described, sex and age of the patients, clinical manifestations at onset, liquor examination, radiographic findings, culture results, urodynamic studies, therapies, and outcome.

The database research produced a total of around 30 cases of MRS. After application of PRISMA guidelines, 2 articles were retrieved, because one described a meningitis caused by Borrelia and the other one reported a case of Vogt-Koyanagi-Harada disease, for a total of 27 cases previously described. Written informed consent from the patients’ legal guardian was required and signed. The study was conducted in accordance with the ethical principles that have their origin in the Declaration of Helsinki.

## Results

Including our case, 29 cases were described in the literature. In the last review, Sakakibara et al. reported 8 MRS cases from 1985 to 2013, with related data regarding sex, age, clinical signs and symptoms, cerebrospinal fluid characteristics, radiological findings, and prognosis. After reviewing the literature to present, we found further 21 MRS cases, including our patient. The features of reported cases of MRS are summarized in Table 1 and Table 2.

**Table 1:** Key clinical and radiologic features of meningitis-retention syndrome patients

Men/women	18/11
Median age at onset, y (range)	13–74
Presence of prodromal symptoms	
Fever, *n* (%)	86%
Headache, *n* (%)	76%
Meningeal symptoms, *n* (%)	65%
Urinary retention onset after prodromes, days (range)	1–10
Nucleated cells/mm^3^ in cerebrospinal fluid (CSF), cells/mm^3^ (range)	24–700 cells/mm^3^
Glucose levels in CSF, mg/dl (range)	26–69 mg/dl
Protein levels in CSF, mg/dL (range)	40–331 mg/dl
PCR performed, *n* (%)	22 (75%)
Patients with positive PCR, *n* (%)	5 (17%)
Patients with isolated pathogen, *n* (%)	7 (24%)
Patients with meningeal enhancement, *n* (%)	5 (17%)
Urodynamic recording performed, *n* (%)	17 (59%)
Patients with detrusor hyporeactivity, *n* (%)	17 (100%)
Patients treated with antivirals, *n* (%)	13 (45%)
Patients treated with antibiotics, *n* (%)	6 (21%)
Patients treated with steroid therapy, *n* (%)	4 (14%)
Urination recovery, weeks (mean)	4.5 weeks

**Table 2:** Review of the previously reported cases of meningitis-retention syndrome

Author, year	Age (years)	Sex	Clinical features and prodromal symptoms			Urinary retention onset after prodroms (days)	Liquor examination			Inflammation index (PCR, WBC)	Isolated pathogen in liquor	MRI findings	Detrusor study	Autoantibodies	Therapy	Urination recovery (weeks)
			Fever	Headache	Meningeal symptoms		Cells	Proteins	Glucose							
Kanno, 1985 [18]	34	F	ND	ND	Yes	ND	40/mm3- 100% L	ND	44 mg/dl	ND	None	ND	Hypoactive	ND	ND	6
Ohe, 1990 [19]	24	F	Yes	Yes	No	ND	312/mm3 - 100% L	260 mg/dl	ND	ND	None	ND	Hypoactive	ND	ND	2
Fukagai, 1996 [20]	46	M	Yes	Yes	No	ND	143/mm3- 81% L	ND	ND	ND	None	ND	Hypoactive	ND	ND	10
Shimizu, 1999–1 [21]	13	M	No	No	Yes	ND	60/mm3- 99% L	45 mg/dl	50 mg/dl	Negative	HSV-2	ND	Hypoactive	ND	ND	5
Shimizu, 1999–2 [21]	18	F	No	No	Yes	ND	109/mm3- 66% L	76 mg/dl	47 mg/dl	Negative	HSV-2	ND	Hypoactive	ND	ND	4
Zenda, 2002 [22]	32	F	Yes	Yes	No	7	370/mm3- 97% L	116 mg/dl	39 mg/dl	Negative	None	ND	Hypoactive	None	ND	10
Sakakibara, 2005–1 [2]	46	M	Yes	Yes	Yes	4	290/mmc3 100% L	80 mg/dl	41 mg/dl	Negative	None	Normal	Hypoactive	ND	Acyclovir + fluconazole	3
Sakakibara, 2005–2 [2]	68	F	Yes	Yes	Yes	1	108/mmc3 100% L	97 mg/dl	41 mg/dl	Negative	None	Normal	Hypoactive	ND	None	2
Sakakibara, 2005–3 [2]	34	M	Yes	Yes	Yes	3	38/mmc3 100% L	71 mg/dl	57 mg/dl	Leukocytosis, normal PCR	None	Normal	Hypoactive	ND	Acyclovir + metilprednisolone	2
Tascilar, 2009 [23]	26	F	Yes	Yes	Yes	10	408/mmc3 100%L	165 mg/dl	38 mg/dl	Negative	None	Enhancement of the leptomeninges of conus medullaris	Hypoactive	ND	Ceftriaxone, ampicillina e acyclovir	8
Lee, 2010 [24]	30	M	Yes	Yes	No	5	439/mmc 90% L	Normal	Normal	Negative	None	Normal	ND	ND	ND	14
Takahashi, 2010 [7]	73	F	Yes	No	Yes	ND	170/mmc3, 43%	95 mg/dl	30 mg/dl	Mild PCR increase	None	Normal	Hypoactive	ND	ND	10
Tateno, 2011 [25]	62	M	Yes	Yes	Yes	7	71/mmc3 100% L	146 mg/dl	56 mg/dl	Negative	None	Normal	Hypoactive	ND	Prednisone	8
Ntziora, 2011 [26]	16	F	Yes	Yes	Yes	6	100/mmc3 100% L	51 mg/dl	46 mg/dl	Negative	None	Normal	ND	ND	Ceftriaxone + acyclovir; ampicillina; gentamycin	2
Krishna, 2012 [27]	50	F	Yes	Yes	Yes	4	700/mmc3, 100 L	150 mg/dl	50 mg/dl	ND	HSV-2	Normal	ND	ND	Acyclovir	2
Cartier, 2014 [28]	24	M	Yes	Yes	Yes	7	94/mmc3 92% L	40 mg/dl	45 mg/dl	Negative	None	Meningeal and medullar enhancement	ND	ND	Ceftriaxone + acyclovir	8
Mankongpaisarnrung, 2013 [11]	26	M	Yes	Yes	Yes	7	225/mmc3 45% L	115 mg/dl	60 mg/dl	Negative	West Nile	Normal	ND	ND	Meropenem + vancomycin + acyclovir	3 days
Basoulis, 2015 [6]	22	M	Yes	Yes	Yes	9	640/mmc3 100%L	180 mg/dl	45 mg/dl	Elevated	None	Normal	Hypoactive	ND	Ceftriaxone + vancomycin	4
Ishii, 2016 [29]	48	M	Yes	Yes	Yes	10	62/mmc3	98 mg/dl	42 mg/dl	Elevated	None	ND	Hypoactive	ND	Acyclovir	2
Shah, 2016 [30]	20	M	Yes	Yes	Yes	10	180/mmc3	75 mg/dl	44 mg/dl	ND	HSV-1	Cerebral and meningeal enhancement	ND	ND	Acyclovir	2
Tanaka, 2017 [31]	29	M	Yes	Yes	No	7	122/mmc3	50 mg/dl	56 mg/dl	Elevated	None	Normal	Hypoactive	ND	ND	2
Hiraga, 2018- 3 patients [32]	21–46 (range)	2 M, 1 F	Yes	Yes	Yes	6–12 (range)	24– 85/mmc3 89% L	64–142 mg/dl (range)	ND	Negative	None	ND	ND	ND	ND	1–3 (range)
Malikova, 2019 [33]	50	F	Yes	Yes	No	ND	512/mmc	64 mg/dl	26 mg/dl	Negative	None	Normal	ND	ND	Acyclovir	1
Suzuki, 2020 [34]	55	M	Yes	No	No	7	143/mmc	121 mg/dl	ND	Negative	EBV	ND	ND	ND	Antiviral and steroids	ND
Sakakibara. 2020 [35]	74	M	No	No	No	0	28/mmc3	44 mg/dl	56 mg/dl	Negative	None	Normal	Hypoactive	Negative	None	No recovery
Kenzaka, 2021 [36]	58	M	Yes	No	No	7	232/mmc	331 mg/dl	69 mg/dl	Negative	VZV	Meningeal enhancement	ND	ND	Acyclovir	11
Our case, 2022	15	M	Yes	Yes	No	6	173/mmc3	158 mg/dl	41 mg/dl	Negative	None	Sacral meningeal enhancement	Hypoactive	Negative	Ceftriaxone, vancomycin, acyclovir, steroids	4

Eighteen of the reported patients were males, with an M:F ratio of 2.3. The age at diagnosis ranged from 13 to 74 years, with a mean age of 36 years. The age at presentation of symptoms was equally distributed for sex. The clinical symptoms and signs frequently described as prodromes were fever (25/29, 86%), headache (22/29, 76%), and meningeal symptoms (19/29, 65%). The mean length of the interval between the onset of the neurological signs and the urinary retention was 6.4 days (range 1–10 days).

The cerebrospinal fluid analysis showed a mild to marked pleocytosis (range 24–700 cells/mm^3^), with increased proteins (range 40–331 mg/dl) and normal glucose levels (range 26–69 mg/dl). The analysis of the common inflammatory indexes (PCR, leukocytosis) was frequently negative, and only in 4 cases they resulted elevated. Apparently, there was no correlation between the elevation of inflammatory indexes and the severity of clinical phenotype or prognosis. In most cases (22/29, 75%), no pathogens were isolated in CSF, while HSV-2 was found in 3 cases and HSV-1, Epstein-Barr virus (EBV), Varicella-Zoster Virus (VZV), and West-Nile Virus in one case each. Herpes viruses turn out to be the most common pathogens associated with MRS, for a total of 6 cases out of 29 (21%). Radiological examination showed only in 5 cases pathological findings, described as marked meningeal enhancement, while in the rest of the cases, MRI was normal. The urodynamic study resulted in a detrusor underactivity in all 17 patients in which a urodynamic recording of the patient’s bladder was performed, including ours. Autoantibodies in CSF were tested only in two patients and resulted negative. Treatment generally consisted in combined therapy with antivirals (13/29), antibiotics (6/29), and steroids (4/29), while only 4 patients were treated without any therapy except for bladder catheterization. The mean period for urination recovery was 4.5 weeks (range 3 days–14 weeks), and only one patient did not completely recover at follow-up.

## Discussion

We present a case of aseptic meningitis (AM) further complicated by urinary retention. AM is a common neurological condition caused by non-bacterial agents (viruses and other pathogens) or by non-infectious diseases (systemic lupus erythematosus, leukemia, lymphoma, and drugs) [3]. The development of urinary retention in the context of AM is known as meningitis-retention syndrome (MRS), firstly described by Sakakibara in 2005 [2], and it is currently considered a self-remitting disease. As in our patient, most of the MRS cases described in the literature presented prodromal symptoms as headache and fever, and the initial examination revealed neurological signs suggestive of sacral nerve dysfunction, including sacral paraesthesia and weakness of the lower extremities [4]. These symptoms are associated with hyporeflexia often mimicking Guillain-Barré syndrome or other polyneuropathies, but in MRS, neurophysiological studies reveal normal nerve conduction, and electromyographic examination is not pathological [4]. In addition, on CSF examination, there is no cytoalbuminologic dissociation, typical of Guillain-Barré syndrome.

In most reported MRS cases, there are no encephalitic symptoms or signs, which distinguish it from ADEM, a rare immune-mediated demyelinating disease involving the central nervous system and characterized by acute onset of multifocal neurological signs [5]. Another distinguishing feature between these two diseases is that MRI of the brain and of the spinal cord reveals no abnormalities in MRS [4], although there has been some speculation regarding reversible cerebral and medullary lesions, as in our case. The principal hypothesis is that MRS could be a mild form of ADEM triggered by a viral infection [4, 6], but one reported that peculiar case is associated with ingestion of two herbal medicines (Shinbu-Tou and Rikkunshi-Tou) by a woman to treat diarrhea, which caused an allergic/autoimmune reaction [7].

Since most cases have been initially treated as meningitis with a broad-spectrum antimicrobial therapy associated with corticosteroids, it is possible that the prompt use of corticosteroids may reduce the inflammation, preventing the cerebral or medullary involvement and the correlated radiological findings, reported in manifest ADEM cases.f1.

Although its pathogenicity is still unclear, MRS seems to have some elements in common with Elsberg syndrome, which is characterized by the combination of acute urinary retention, constipation, erectile dysfunction, herpetic genital vesicle, lumbosacral radicular pain, hypoesthesia, and muscle weakness [8]. It was first described in 1913 as lumbosacral radiculopathy, with acute urinary retention secondary to lumbosacral myeloradiculitis, caused by a viral infection such as HSV-2, HHV-6, and *Angiostrongylus cantonensis* infection [9]. In Elsberg syndrome, urinary retention is due to the reactivation of HSV in the sacral dorsal root ganglia with axonal spread to the spinal cord. This can usually be visualized as hyperintense T2 lesions on spinal MRI, not common in MRS cases [10]. In our case, HSV 1–2 PCR on CSF and blood were both negative, thus ruling out this diagnostic hypothesis.

As in our case, other causes of aseptic meningitis are usually ruled out based on the anamnestic data, the negativity of culture or molecular tests, and immunological results.

MRS, CSF, blood, and urine cultures are negative in most cases, and no cause is determined.

When a pathogen has been isolated, it has always been a Herpesvirus except for a case of West Nile MRS [11]. These data confirmed the correlation between MRS and Elsberg syndrome and the possible clinical and microbiological overlap. Thus, the absence of spinal involvement at MRI is a diriment for differential diagnosis between them.

Considering the negative cultures in most of the patients, we may speculate the autoimmune etiology of MRS, sustaining the hypothesis that MRS could be a mild form of ADEM without medullary involvement, due to prompt use of corticosteroids and antiviral that reduce the inflammation and treat the infection, avoiding the detection of the pathogen.

In most published cases, CSF analysis revealed mild to severe lymphocytic pleocytosis, increased protein content, and normal to mildly decreased glucose content in all patients [4].

Increased myelin basic protein (MBP), suggestive of central nervous system demyelination, is reported in one patient by Sakakibara et al., but it was never tested in others [4]. This result, even if occasional, may further support the hypothesis that MRS is a mild variant of ADEM, which selectively affects the lower urinary tract (LUT) innervation.

Adenosine deaminase (ADA) is detected in the CSF of two patients. The CSF ADA estimation appeared useful for establishing a diagnosis of tuberculous meningitis [12]. Although non-tuberculous meningitis could raise the CSF ADA levels, non-infectious neurological diseases do not commonly increase it [13].

Nevertheless, increased ADA levels were reported in the CSF of a patient affected by autoimmune glial fibrillary acidic protein (GFAP) astrocytopathy (GFAP-A), a rare disease sustained by anti-GFAP antibodies, usually presenting as an acute disorder, characterized by myelitis, abnormal vision, ataxia, altered consciousness, and seizures. A history of symptoms of upper respiratory tract infection is found in 40% of the GFAP-A cases. [14] Although the suggestive history of infection, due to clinical differences between GFAP-A and MRS, the serological detection of ADA in 2 patients with MRS seems occasional.

Therefore, CSF findings in MRS are suggestive of nonspecific meningeal inflammatory involvement, and autoantibody detection is necessary. In our case, IgM anti-GM1 and IgM anti-GM2 were detected in serum so as to rule out Guillain-Barrè syndrome and chronic inflammatory demyelinating polyneuropathy (CIDP), respectively [15, 16]. Our results may be considered a nonspecific sign of demyelination, but the role of autoantibodies is still unclear.

When performed, the urodynamic study results have shown that most of the patients reported had an areflexic detrusor, which results in an inability to contract the bladder properly on voiding [2]. Several hypotheses have been postulated to explain the detrusor hypofunction and urinary retention in MRS. Central nervous system lesions that affect the spinal cord or the brain may cause detrusor areflexia, which is common in patients with transverse myelitis or ADEM [17], but, as described above, encephalitic and myelitic features are absent in patients with MRS. We think that urinary retention in MRS has a neurologic etiology, since none of the reported cases, including our patient, had urologic abnormalities such as urinary tract infection, and there was a strong chronological association in that the urinary retention appeared simultaneously or just after the occurrence of aseptic meningitis. However, the lesion site responsible for urinary retention in MRS remains obscure, but we hypothesize that a meningeal irritation may lead to an initial acute spinal shock, which may compromise LUT innervation.

MRS is believed to be a self-limited disease, and no evidence suggests that any treatment affects its clinical course. Although immune treatments such as steroids, antibiotics, and antiviral treatment have been tried in most patients, their effectiveness remains unclear. In the literature, cases described were treated with different therapeutic combinations, and there is no correlation between a specific therapy and length of hospitalization. These findings support the idea that MRS is a self-remitting condition and only supportive therapy may be necessary.

## Conclusion

In summary, MRS is an uncommon syndrome characterized by aseptic meningitis and acute urinary retention with exclusive involvement of leptomeninges. It may represent a mild form of ADEM, without radiological detectable medullary involvement, probably due to the prompt use of corticosteroids. The management of MRS includes the prevention of bladder injury from overdistension with the use of an indwelling catheter.

Further studies are needed to better explain the mechanism behind this syndrome and evaluate the effectiveness of corticosteroids in reducing the period of urinary retention.

## Data Availability

Data available on request due to privacy/ethical restrictions.
